# The burden of rare cancers among adults in the Canton of Geneva, Switzerland, from 2011 to 2020

**DOI:** 10.3389/fonc.2025.1557424

**Published:** 2025-04-07

**Authors:** Nathalie Bot, Evelyne Fournier, Marie-Laure Amram, Laura Botta, Alice Bernasconi, Elisabetta Rapiti

**Affiliations:** ^1^ Institute of Global Health, Faculty of Medicine, University of Geneva, Geneva, Switzerland; ^2^ Geneva Cancer Registry, University of Geneva, Geneva, Switzerland; ^3^ Clinique et Permanence d’Onex, Geneva, Switzerland; ^4^ Evaluative Epidemiology Unit, Department of Epidemiology and Data Science, Fondazione IRCCS “Istituto Nazionale dei Tumori”, Milan, Italy

**Keywords:** rare cancer, cancer registry, population-based study, burden, incidence, survival, molecular tumorboard

## Abstract

**Introduction:**

Globally, cancer cases are expected to significantly increase due to population growth and aging, reaching 29.9 million by 2040 (+49.5% since 2022) and 32.6 million by 2045 (+63%), with countries like Switzerland forecasting a 36.5% increase. Rare cancers, defined as less than six cases/100,000 individuals/year, account for 15-24% for recent nationwide studies but they have fewer treatment options and lower survival rates. Using the Geneva Cancer Registry, we analyzed rare cancer incidence and survival rates in adults from the canton of Geneva, Switzerland (2011–2020), with the aim of informing future research at local and national levels.

**Methods:**

We analyzed adult patients diagnosed with invasive cancers (2011–2020) in Geneva using Geneva Cancer Registry data, which were annually updated. Rare cancers were defined according to RARECAREnet criteria (incidence less than six cases/100,000 individuals/year) and categorized into Tier 1 and Tier 2 entities based on clinical features. Crude and standardized incidence rates were calculated for both sexes using the 1976 European reference population, as well as age-specific rates for rare and common cancers. Five-year survival rates were estimated using the Kaplan–Meier method. Survival differences between rare and common cancers were assessed using log-rank tests and Cox proportional hazards models adjusted for age and gender. Statistical analyses were performed using STATA software.

**Results:**

Between 2011 and 2020, 31,233 invasive cancers were diagnosed in adults in Geneva, of which 4,296 cases (13.75%) were classified as rare based on aforementioned thresholds. While some rare Tier 1 cancers included common subtypes, most Tier 2 cancers (141 in total) were classified as rare, with significant gender disparities. Men had higher rare cancer rates such as epithelial hypopharynx, larynx, and liver tumors, while women had higher rates of squamous cell carcinoma of the anus. Rare neuroendocrine tumors, central nervous system tumors, and hematological malignancies, such as follicular B lymphoma and acute myeloid leukemia, were also prevalent among rare cancers. Rare cancers increase with age, but less so than common cancers. The 5-year survival rate for rare cancers was 58.4% when compared with 62.3% for common cancers, indicating a 15.7% higher risk of death for patients with these cancers.

**Discussion:**

These findings highlight the critical challenges and requirements of targeted research and improving care strategies for rare cancers. Efforts combatting such cancers include European Reference Networks and the Swiss Sarcoma Network, which have improved access to care via collaborative efforts. In Switzerland, Molecular Tumor Boards have leveraged genomic knowledge to refine treatments and allow patient participation in clinical trials. Early referral to such boards for aggressive or treatment-limited cancers can streamline care and facilitate patient access to specialist centers. However, Switzerland requires more comprehensive data on the distribution of rare cancers in terms of age, gender, and region to improve management strategies at national levels.

## Introduction

Focusing exclusively on global population growth and aging, population-based projections from 185 countries (GLOBOCAN project) indicate that the total number of global cancer cases will increase to 29.9 million by 2040 (+49.5% since 2022) and to 32.6 million (+63%) by 2045. In countries with a very high human development index, such as Switzerland, an increase of 36.5% is expected, which corresponds to approximately +3.4 million individuals (1).

By definition, rare cancers have low incidence rates in the general population, i.e. < 6 cases/100,000 individuals/year. However, overall, rare cancers account for a significant proportion of all cancer cases; according to the RARECAREnet working group, the overall incidence of rare cancers in Europe is approximately 20%–24% of total cancer incidences (2, 3), and the range extends from 15% to 24% if we examine recent national studies in and outside Europe (3–10).

Given limited therapeutic options for many rare cancers and their low survival rates when compared to common cancers, robust population data on incidences, survival, and trends are required to fully characterize this burden on society (11).

Our main objective was to provide a comprehensive overview of rare cancer incidences and survival rates in adults in the Canton of Geneva over the last decade (2011–2020) using the Geneva Cancer Registry database. This work provides a platform for further studies investigating the rare cancer burden in Geneva and in Switzerland.

## Methods

### Study population and general authorization to collect data

Patients were selected from the Geneva Cancer Registry; all were aged ≥ 18, diagnosed with invasive cancer between 2011 and 2020, and were resident in the Canton of Geneva at the time of diagnosis. Vital status was updated annually by cross-referencing with Cantonal Population Office data.

The Geneva Cancer Registry has a general authorization mandate to collect nominative data and analyze anonymized data. The Experts Commission (Generalbewilligung), Ethics Committees, and the National Law on Cancer Registration authorize the collection of risk and prognostic factors from cancer cases without a need for individual consent. Patients are informed of the Registry but can refuse access to their data by the Registry. Registry management confirms that no health-related personal data and biological materials are used if an individual provides a written rejection reflecting this.

Our study protocol met the regulatory requirements of the Swiss Federal Human Research Act and the Human Research Ordinance. The protocol was also submitted to a local Ethics Committee (Reference Number; 2023-01420), which indicated the project was not subjected to authorization.

### Rare cancer definition and classification

We applied RARECAREnet criteria, which defines rare cancers using a crude incidence of < 6 cases/100,000 individuals/year. Tumors were classified as Tier 1 and Tier 2 according to Botta et al. (1). More specifically, each major rare cancer family (i.e. head and neck cancers, digestive tract cancers, etc.) were sub-classified into Tier 1 entities (e.g., epithelial stomach tumors) and then into distinct smaller cancer groups or Tier 2 entities. Tiers corresponded to clinically relevant topography and morphology information (e.g., ‘squamous cell carcinoma of the stomach’) (12).

### Statistical analyses

For each rare cancer site in our analysis period, for both sexes, we calculated crude and standardized incidence rates per 100,000 individuals using the standard 1976 European reference population. We calculated age-specific incidence rates for both groups (Tiers 1 and 2) for rare and common (> 6 cases/100,000 individuals) cancers. We calculated 95% confidence intervals (CIs) based on the hypothesis that rare cancers followed a Poisson distribution. Five-year survival rates were calculated as the time between the initial diagnosis date and the date of death from any cause, emigration, or end of follow-up, whatever came first, using the Kaplan–Meier method. The cut-off survival date was December 31^st^, 2023. To compare survival rates between rare and common cancers, we used log-rank tests. A Cox proportional hazards regression model was performed to assess the 5-year risk of death among patients with rare cancer, adjusting for age and gender. Risk proportionality assumption was tested on the basis of Schoenfeld residuals analysis after fitting the multivariate Cox model. Statistical analyses were performed using STATA SE18 (College Station, TX, USA).

## Results

All rare adult cancer cases observed in the canton of Geneva (2011–2020) are shown in **Supplementary Table S1** (**Supplementary Materials**). As outlined, RARECAREnet applied a crude incidence threshold of < 6 cases/100,000 individuals/year, below which a cancer is considered a rare clinical entity. In solely considering Tier 1 crude incidence rates, it appeared that several cancers could be defined as common as they had a crude incidence of > 6 cases/100,000 individuals. For example, in the rare digestive cancer family, epithelial stomach, esophagus, colon, rectum, pancreas, liver, and intraepatic bile tract tumors were all common. However, Tier 1 entities may also include rare Tier 2 entities. To highlight Tier 2 cancer incidence rates in our list, we labeled cancers that had an incidence of < 6/100,000 as “R” (Rare). Consequently, most Tier 2 entities, 141 in total, were classified as rare and corresponded to 4,296 cases. Tier 2 cancers, not labeled with “R”, were otherwise recognized as common, such as adenocarcinoma of the colon or pancreas. Even when the R was slightly over the 6/100,000 cut-off, cancers were not labeled as rare as these results were used as viable comparators with other European standards (2).

Stratifying Tier 2 entities by sex (**Supplementary Table S1**) indicated that the incidence of several cancer subtypes showed significant disparities between the sexes, often exceeding 6/100,000 cases in men while lower in women. For instance, in head, neck, and digestive tract cancer families, epithelial hypopharynx, larynx, oropharynx or oral cavity and lip tumors, epithelial esophagus tumors, and epithelial liver and digestive tract tumors showed significantly lower crude rates in women when compared with men. Conversely, squamous cell carcinoma (SCC) of the anus was observed in 132 women versus 57 in men (crude rate of 6.39 in women versus 3.01 in men).

Overall, between 2011 and 2020, 31,233 invasive cancer cases were diagnosed in the adult population in Geneva. When considering cases with incidence rates of < 6/100,000 individuals/year, our cases accounted for 4,296 in total. Of these, 2,185 cases occurred in men and 2,111 in women, corresponding to 13.75% of the total cancer landscape in Geneva over the last decade.

**Figure 1:**
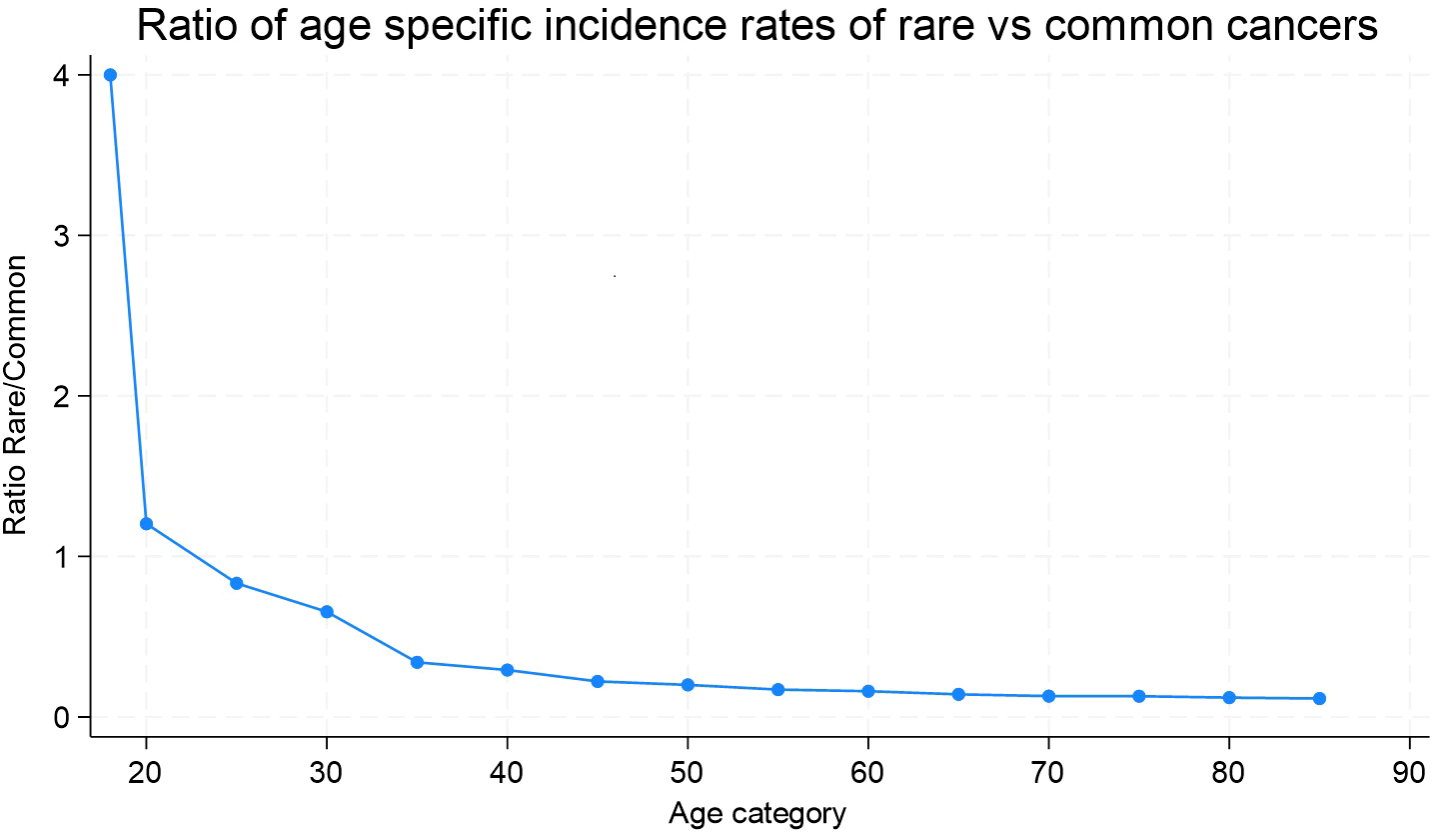
Ratio of age-specific incidence rates of rare versus common cancers. Geneva, 2011-2020, ages 18 and more.

As shown in **Figure 1**, rare cancers are predominant in the first two groups of age, 18-19 and 20-24 years. After age 24, we observe a constant decrease in the ratio of rare to common cancer incidence rates, suggesting that common cancers become disproportionately more frequent with aging. This trend continues steadily, with a slight plateau around age 70. The incidence rates of several Tier 2 entities, while labeled as rare, showed higher crude rates within their cancer family. Among these cancers specific to the sexes, we observed that testicular and paratesticular carcinomas and epithelial tumors of the pelvis and ureter predominated in men, with crudes rates of 3.86 for non seminomatous testicular carcinoma, 5.7 for seminomatous carcinoma, and 3.59 for transitional cell carcinoma of the pelvis and ureter. In women, epithelial tumor incidence rates in the cervix uteri were notable (crude rate; 3.87 for SCC and 1.55 for adenocarcinoma (AC)), as were those in the vulva and vagina (crude rate; 3.15 for SCC).

The notable prevalence of rare neuroendocrine cancers, particularly well-differentiated, non-functioning endocrine pancreas and digestive tract carcinomas, was observed in both sexes (crude rates; 5.8 and 4.49 in women and men, respectively). In terms of central nervous system tumors, astrocytic tumors showed a gender disparity with a crude rate of 8.3 in men versus 4.26 in women.

Finally, among rare hematological malignancies, four rare cancer types had higher incidences across the sexes: Follicular B lymphoma, with 86 cases in women (crude rate; 4.16) and 84 cases in men (crude rate; 4.4); Hodgkin lymphoma, with crude rates of 2.95 and 4.28 in women and men, respectively; Other myelodysplastic syndromes were rare in women (crude rate; 5.71) but not in men (crude rate; 9.30). Acute myeloid leukemia, the fourth subtype, was represented by a significant cohort with 99 cases in women (crude rate; 4.79) and 102 in men (crude rate; 5.39).

**Figure 2:**
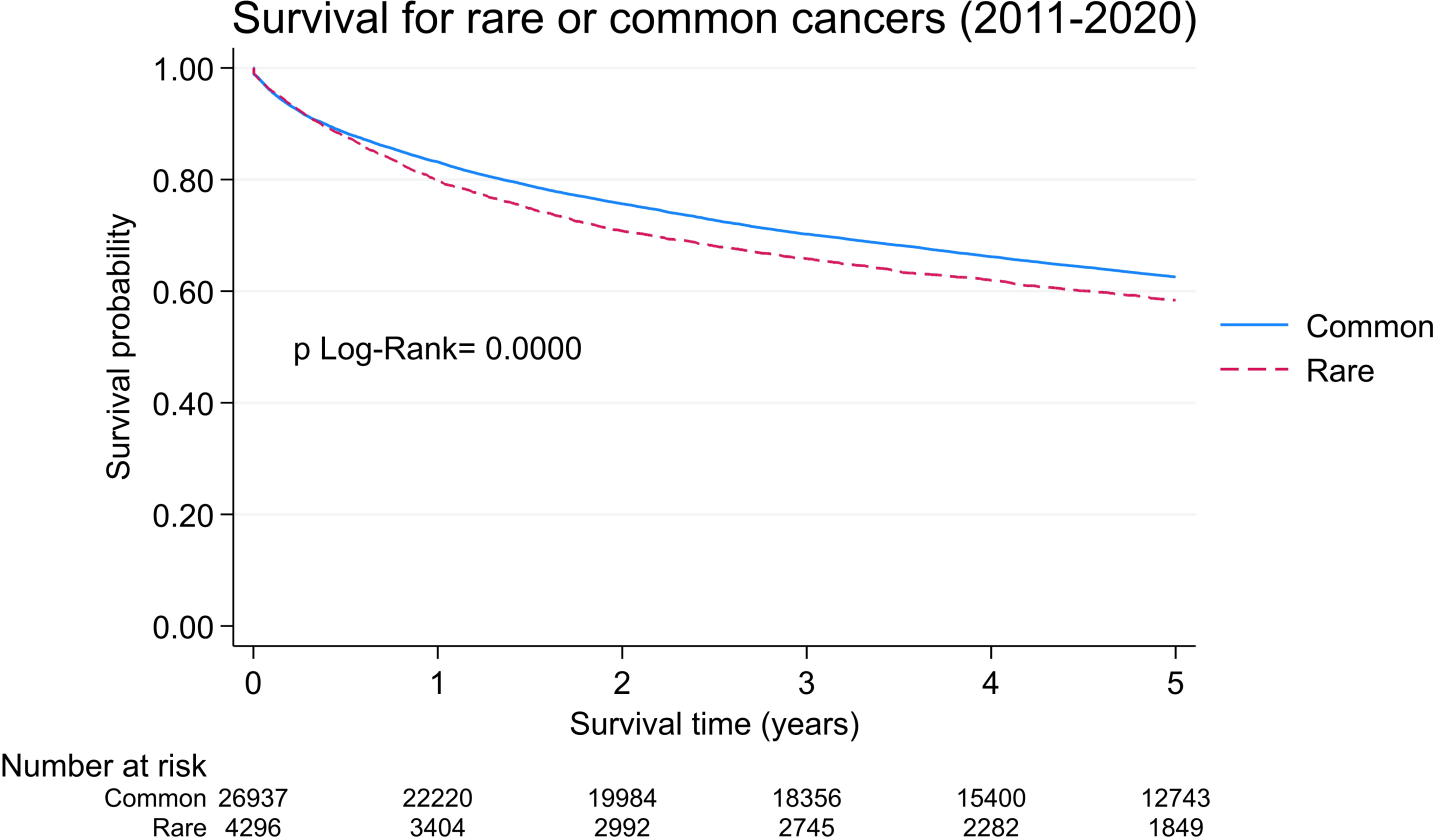
Five- year overall survival probabilities for rare and common cancers. Geneva, 2011-2020, age 18 and more.

The 5-year survival probability for patients diagnosed with a rare cancer was 58.4% (95% CI: 56.8–59.9), while for patients with common cancers, this was 62.3% (95% CI: 62.0–63.2) (p log-rank test <0.001), corresponding to an increased 15.7% (p <0.001, Cox regression model) risk of dying from a rare cancer vs. a common cancer (**Figure 2**). When adjusting the Cox model for age and sex, the mortality risk of rare cancers worsens further in comparison to common cancers (HR = 1.40, 95% CI: 1.33-1.48, p < 0.001) (**Table 1**).

**Table 1:** Multivariate Cox proportional hazard model of 5-year survival.

	Hazard Ratio (95% CI)	p
Age group		
<50	Ref	
50-69	2.36 [2.13-2.62]	**<0.001**
70+	5.36 [4.85-5.92]	**<0.001**
Gender		
Men	Ref	
Women	0.85 [0.82-0.88]	**<0.001**
Commun or rare cancer		
Commun	Ref	
Rare	1.40 [1.33-1.47]	**<0.001**

p-Value : Statistically significant values are indicated in bold.

## Discussion

In the Canton of Geneva between 2011 and 2020, 4,296 rare adult cancer cases were diagnosed, accounting for more than 13.75% of all cancer diagnoses during this period. Rare cancers from the head and neck, digestive tract, male genital and urogenital regions, and hematological cancer families, showed the highest crude incidence rates. Sex-specific differences were observed, with a clear predominance for men among subtypes, with the exception of anal cancer which was more prevalent in women. Although these analyses are exploratory and need to be confirmed by larger databases, an analysis performed using a 0.01 significancy threshold to take into account multiple testing, yielded the same results.

Notably, age-specific rare cancer incidence rates increased less steeply with age when compared with common cancers. Finally, within 5 years of diagnosis, patients diagnosed with a rare cancer would have a higher risk of death than patients in the common cancer group.

The rare cancer percentage (13.75%) identified in our study was slightly lower than that reported in recent nationwide studies, which ranged from 15% to 24% (3–10). However, differences in rare cancer definitions and incidence thresholds have been recorded between North American and European classifications, which may influence percentages, as described by Walker et al. (7). Additionally, study population differences may have contributed to these discrepancies: we focused exclusively on adults, while most aforementioned studies included pediatric populations. In the North American context, Hofer et al. highlighted this and showed increased rare cancer numbers when pediatric cases were included (9). Also, the population at risk in our study was small when compared with population sizes in national or international studies (3–10).

Our data indicated that a significant proportion of rare cancers among adults occurred in certain rare cancer families, notably cancers of the head and neck, digestive, genital and urogenital regions, and hematological malignancies. A recent European study examining rare solid cancers in adults, conducted in 29 European Union member states between 2006 and 2013, also highlighted predominant incidence rates (crude and age-standardized) for rare head and neck cancers, digestive cancers (gallbladder and extrahepatic bile duct), and male genital and urogenital cancers (testicular and pelvic cancers) (13). Moreover, in the same study, sex-specific differences were recorded, indicating a male predominance for head and neck, digestive, and urological cancers. In contrast, there was a clear female predominance for thyroid, gallblader and extrahepatic biliary tract and anal canal cancers.

Several population-based studies have shown that the incidence rates of these rare cancer subtypes increase with age (14–17). Such increased incidence rates are probably sustained not only by demographic changes, such as population growth and aging, but also by underlying epidemiological risks (18). Indeed, unlike childhood and adolescent cancers, most of these cancers are associated with common lifestyle risk factors, including smoking, alcohol consumption, dietary, and sexual habits (14, 19–21). This latter point may explain the higher cancer prevalence observed in men in our study, as it reflected the global cancer burden among men estimated by GLOBOCAN 2022, which reported a higher cancer burden due to modifiable risk factors in men (22).

## Perspectives

Rare cancers impose significant burdens on our healthcare systems, which are represented by diagnosis delays, limited treatment options, and sparse research initiatives, culminating in lower survival rates. In Geneva, we observed lower rare cancer survival rates when compared with common cancer rates (**Figure 2**), which unfortunately was consistent with survival rate data from the USA (5) and Europe (3, 8). In 2017, such disparity outcomes led to the creation of European Reference Networks (ERNs); this gave rise to ERN EURACAN (Rare Adult CANcer) which sought to improve the quality of care for patients with rare adult cancers by providing better care access (23). In practice, adult patients with rare cancers can be referred to one of these reference centers (106 in total) across 25 European countries (24). In Switzerland, the Swiss Sarcoma network, which focuses on sarcoma, has established a partnership with EURACAN.

Since 2016, in French-speaking Switzerland, patients with complex or rare and/or refractory cancers have had access to cutting-edge precision oncology resources via the Romand Oncology Network (25). Precision oncology access via Molecular Tumor Boards (MTBs) has also provided patients with the latest genomic and transcriptomic technologies as well as the latest biomedical knowledge and clinical practices. MTBs bring together multidisciplinary expert groups who collaboratively analyze and interpret patients’ molecular tumor profiles to optimize treatment strategies by identifying biomarkers (26). Depending on the molecular profile of a patient’s tumor, a clinical trial may also be proposed, either at a university hospital in Switzerland or abroad. This approach can streamline the disease course by centralizing the complex analysis and interpretation of a patient’s tumor while decentralizing treatment protocols. Within this framework, certain rare cancer subtypes merit immediate referral to an MTB, rather than waiting for later disease stages when conventional treatment options have been exhausted. For example, this approach can include cancers in rare locations (the thymus and parathyroid glands), cancers with unusual histology in common locations (breast sarcoma), cancers with aggressive histology but few therapeutic options (poorly differentiated neuroendocrine carcinoma), or cancers with limited treatment alternatives (mesothelioma or adrenocortical carcinoma). For these rare cancers, the MTB platform may represent the first phase in the care trajectory, allowing rare cancer patients to benefit from cutting-edge oncology and expertise. This approach not only avoids inefficient and unnecessary treatments, but facilitates rare cancer patient referral to specialized reference centers when possible.

## Conclusions

In recent years, several national or international epidemiological and specific rare cancer studies have been conducted using cancer registry data, including Swiss data (14, 17, 19, 27). However, in Switzerland, the rare cancer burden in adults is not known and a detailed picture of this distribution by sex and age across Swiss cantons and/or linguistic regions remains unclear. Characterizing this burden is an important step toward implementing national and regional rare cancer management care in Switzerland.

## Data Availability

The original contributions presented in the study are included in the article/**Supplementary Material**. Further inquiries can be directed to the corresponding author.

## Supplementary material

Supplementary Table 1 Rare cancer incidence rates in adults and by sex (2011– 2020) in Geneva, Switzerland.

Supplementary Table 2 Standardized incidence rates for men and women (2011–2020) in Geneva, Switzerland.
