# Supplementary material for: The burden of rare cancers among adults in the Canton of Geneva, Switzerland, from 2011 to 2020
Source: Front Oncol. 2025 Apr 7;15:1557424. doi: 10.3389/fonc.2025.1557424 (PMC12009699; doi:10.3389/fonc.2025.1557424)
Supplement: Supplementary Table 1 — Rare cancer incidence rates in adults and by sex (2011– 2020) in Geneva, Switzerland. [file Table1.docx]

| Supplementary Table 1. Incidence rates for rare cancers in all adults, and by sex, from 2011-2020 in Geneva, Switzerland | | | | | | | | | | |  |
| --- | --- | --- | --- | --- | --- | --- | --- | --- | --- | --- | --- |
| **Cancer sites** | **Total adult population** | | | |  | **Men** | |  | **Women** | |  |
|  | **Rare** | **N** | **Crude rate** | **95% CI** |  | **N** | **Crude rate** |  | **N** | **Crude rate** |  |
| **Head and neck*** |  | **425** |  |  |  | **309** |  |  | **116** |  |  |
| **EPITHELIAL TUMOURS OF NASAL CAVITY AND SINUSES **** |  |  |  |  |  |  |  |  |  |  |  |
| Squamous cell carcinoma with variants of nasal cavity and sinuses******* | **R** | 27 | 0.68 | [0.16- 1.77] |  | 14 | 0,74 |  | 13 | 0,63 |  |
| Undifferentiated carcinoma of nasal cavity and sinuses | **R** | 1 | 0.03 | [0.00- 0.58] |  | 1 | 0,05 |  | 0 | 0,00 |  |
| **EPITHELIAL TUMOURS OF NASOPHARYNX** |  |  |  |  |  |  |  |  |  |  |  |
| Squamous cell carcinoma with variants of nasopharynx | **R** | 19 | 0.48 | [0.08- 1.44] |  | 15 | 0,79 |  | 4 | 0,19 |  |
| **EPITHELIAL TUMOURS OF MAJOR SALIVARY GLANDS AND SALIVARY-GLAND TYPE TUMOURS** | | |  |  |  |  |  |  |  |  |  |
| Epithelial tumour of major salivary glands | **R** | 64 | 1.62 | [0.69- 3.11] |  | 34 | 1,80 |  | 30 | 1,45 |  |
| Salivary gland type tumour of head and neck | **R** | 20 | 0.51 | [0.09- 1.749] |  | 7 | 0,37 |  | 13 | 0,63 |  |
| **EPITHELIAL TUMOURS OF HYPOPHARYNX AND LARYNX** |  |  |  |  |  |  |  |  |  |  |  |
| Squamous cell carcinoma with variants of hypopharynx | **R** | 98 | 2.47 | [1.27- 4.24] |  | 80 | 4,23 |  | 18 | 0,87 |  |
| Squamous cell carcinoma with variants of larynx | **R** | 164 | 4.14 | [2.51- 6.34] |  | 135 | 7,13 |  | 29 | 1,40 |  |
| **EPITHELIAL TUMOURS OF OROPHARYNX** |  |  |  |  |  |  |  |  |  |  |  |
| Squamous cell carcinoma with variants of oropharynx |  | 284 | 7.17 | [4.94- 9.97] |  | 204 | 10,78 |  | 80 | 3,87 |  |
| **EPITHELIAL TUMOURS OF ORAL CAVITY AND LIP** |  |  |  |  |  |  |  |  |  |  |  |
| Squamous cell carcinoma with variants of oral cavity |  | 240 | 6.06 | [4.03- 8.657] |  | 153 | 8,08 |  | 87 | 4,21 |  |
| Squamous cell carcinoma with variants of lip | **R** | 30 | 0.76 | [0.20- 1.88] |  | 21 | 1,11 |  | 9 | 0,44 |  |
| **EPITHELIAL TUMOURS OF TRACHEA** |  |  |  |  |  |  |  |  |  |  |  |
| Squamous cell carcinoma with variants of trachea | **R** | 1 | 0.03 | [0.00- 0.58] |  | 1 | 0,05 |  | 0 | 0,00 |  |
| Salivary gland type tumour of trachea | **R** | 1 | 0.03 | [0.00- 0.58] |  | 1 | 0,05 |  | 0 | 0,00 |  |
| **Rare digestive** |  | **798** |  |  |  | **421** |  |  | **377** |  |  |
| **EPITHELIAL TUMOURS OF OESOPHAGUS** |  |  |  |  |  |  |  |  |  |  |  |
| Squamous cell carcinoma with variants of oesophagus | **R** | 188 | 4.75 | [2.98- 7.08] |  | 117 | 6,18 |  | 71 | 3,44 |  |
| Adenocarcinoma with variants of oesophagus | **R** | 102 | 2.58 | [1.34- 4.37] |  | 82 | 4,33 |  | 20 | 0,97 |  |
| Salivary gland type tumour of oesophagus | **R** | 1 | 0.03 | [0.00- 0.58] |  | 0 | 0,00 |  | 1 | 0,05 |  |
| Undifferentiated carcinoma of oesophagus | **R** | 3 | 0.08 | [0.00- 0.70] |  | 2 | 0,11 |  | 1 | 0,05 |  |
| **EPITHELIAL TUMOURS OF STOMACH** |  |  |  |  |  |  |  |  |  |  |  |
| Squamous cell carcinoma with variants of stomach | **R** | 6 | 0.15 | [0.00- 0.86] |  | 4 | 0,21 |  | 2 | 0,10 |  |
| Supplementary Table 1. Cont. Incidence rates for rare cancers in all adults, and by sex, from 2011-2020 in Geneva, Switzerland | | | | | | | | | | |  |
| **Cancer sites** | **Total adult population** | | | |  | **Men** | |  | **Women** | |  |
|  | **Rare** | **N** | **Crude rate** | **95% CI** |  | **N** | **Crude rate** |  | **N** | **Crude rate** |  |
| Undifferentiated carcinoma of stomach | **R** | 3 | 0.08 | [0.00- 0.70] |  | 1 | 0,05 |  | 2 | 0,10 |  |
| **EPITHELIAL TUMOURS OF SMALL INTESTINE** |  |  |  |  |  |  |  |  |  |  |  |
| Adenocarcinoma with variants of small intestine | **R** | 45 | 1.14 | [0.40- 2.44] |  | 24 | 1,27 |  | 21 | 1,02 |  |
| **EPITHELIAL TUMOURS OF COLON (including appendix)** |  |  |  |  |  |  |  |  |  |  |  |
| Adenocarcinoma with variants of colon |  | 1563 | 39.47 | [33.88- 45.63] |  | 782 | 41,30 |  | 781 | 37,79 |  |
| Squamous cell carcinoma with variants of colon | **R** | 1 | 0.03 | [0.00- 0.58] |  | 0 | 0,00 |  | 1 | 0,05 |  |
| Fibromixoma and low grade mucinous adenocarcinoma (pseudomixoma peritonei) of the appendix | **R** | 19 | 0.48 | [0.08- 1.44] |  | 9 | 0,48 |  | 10 | 0,48 |  |
| **EPITHELIAL TUMOURS OF RECTUM** |  |  |  |  |  |  |  |  |  |  |  |
| Adenocarcinoma with variants of rectum |  | 512 | 12.93 | [9.85- 16.58] |  | 284 | 15,00 |  | 228 | 11,03 |  |
| Squamous cell carcinoma with variants of rectum | **R** | 10 | 0.25 | [0.01- 1.05] |  | 2 | 0,11 |  | 8 | 0,39 |  |
| **EPITHELIAL TUMOURS OF ANAL CANAL** |  |  |  |  |  |  |  |  |  |  |  |
| Squamous cell carcinoma with variants of anal canal | **R** | 189 | 4.77 | [3.00- 7.11] |  | 57 | 3,01 |  | 132 | 6,39 |  |
| Adenocarcinoma with variants of anal canal | **R** | 7 | 0.18 | [0.00- 0.91] |  | 3 | 0,16 |  | 4 | 0,19 |  |
| Paget's disease of anal canal | **R** | 1 | 0.03 | [0.00- 0.58] |  | 1 | 0,05 |  | 0 | 0,00 |  |
| **EPITHELIAL TUMOURS OF PANCREAS** |  |  |  |  |  |  |  |  |  |  |  |
| Adenocarcinoma with variants of pancreas |  | 600 | 15.15 | [11.79- 19.08] |  | 315 | 16,64 |  | 285 | 13,79 |  |
| Squamous cell carcinoma with variants of pancreas | **R** | 2 | 0.05 | [0.00- 0.64] |  | 2 | 0,11 |  | 0 | 0,00 |  |
| Acinar cell carcinoma of pancreas | **R** | 6 | 0.15 | [0.00- 0.86] |  | 4 | 0,21 |  | 2 | 0,10 |  |
| Intraductal papillary mucinous carcinoma invasive of pancreas | **R** | 2 | 0.05 | [0.00- 0.64] |  | 1 | 0,05 |  | 1 | 0,05 |  |
| **EPITHELIAL TUMOURS OF LIVER AND INTRAEPATIC BILE TRACT (IBT)** |  |  |  |  |  |  |  |  |  |  |  |
| Hepatocellular carcinoma of Liver and IBT |  | 321 | 8.11 | [5.72- 11.06] |  | 263 | 13,89 |  | 58 | 2,81 |  |
| Hepatocellular carcinoma, fibrolamellar | **R** | 1 | 0.03 | [0.00- 0.58] |  | 0 | 0,00 |  | 1 | 0,05 |  |
| Cholangiocarcinoma of IBT | **R** | 56 | 1.41 | [0.56- 2.83] |  | 34 | 1,80 |  | 22 | 1,06 |  |
| Adenocarcinoma with variants of liver and IBT | **R** | 25 | 0.63 | [0.14- 1.69] |  | 14 | 0,74 |  | 11 | 0,53 |  |
| Undifferentiated carcinoma of liver and IBT | **R** | 2 | 0.05 | [0.00- 0.64] |  | 2 | 0,11 |  | 0 | 0,00 |  |
| Squamous cell carcinoma with variants of liver and IBT | **R** | 2 | 0.05 | [0.00- 0.64] |  | 2 | 0,11 |  | 0 | 0,00 |  |
| **EPITHELIAL TUMOURS OF GALLBLADDER AND EXTRAHEPATIC BILIARY TRACT (EBT)** | |  |  |  |  |  |  |  |  |  |  |
| Adenocarcinoma with variants of gallbladder | **R** | 32 | 0.81 | [0.22- 1.96] |  | 7 | 0,37 |  | 25 | 1,21 |  |
| Supplementary Table 1. Cont. Incidence rates for rare cancers in all adults, and by sex, from 2011-2020 in Geneva, Switzerland | | | | | | | | | | |  |
| **Cancer sites** | **Total adult population** | | | |  | **Men** | |  | **Women** | |  |
|  | **Rare** | **N** | **Crude rate** | **95% CI** |  | **N** | **Crude rate** |  | **N** | **Crude rate** |  |
| Adenocarcinoma with variants of EBT | **R** | 94 | 2.37 | [1.20- 4.11] |  | 52 | 2,75 |  | 42 | 2,03 |  |
| **PANCREATOBLASTOMA** |  |  |  |  |  |  |  |  |  |  |  |
| Pancreatoblastoma | **R** | 1 | 0.03 | [0.00- 0.58] |  | 1 | 0,05 |  | 0 | 0,00 |  |
| **Rare thoracic** |  | **459** |  |  |  | **262** |  |  | **197** |  |  |
| **EPITHELIAL TUMOURS OF LUNG** |  |  |  |  |  |  |  |  |  |  |  |
| Squamous cell carcinoma with variants of lung |  | 508 | 12.83 | [9.76- 16.47] |  | 345 | 18,22 |  | 163 | 7,89 |  |
| Adenocarcinoma with variants of lung |  | 1270 | 32.07 | [27.06- 37.65] |  | 655 | 34,60 |  | 615 | 29,76 |  |
| Adenosquamous carcinoma of lung | **R** | 13 | 0.33 | [0.03- 1.19] |  | 8 | 0,42 |  | 5 | 0,24 |  |
| Large cell carcinoma of lung | **R** | 10 | 0.25 | [0.01- 1.05] |  | 5 | 0,26 |  | 5 | 0,24 |  |
| Poorly differentiated endocrine carcinoma of lung |  | 353 | 8.91 | [6.40- 12.00] |  | 195 | 10,30 |  | 158 | 7,65 |  |
| Salivary gland type tumour of lung | **R** | 5 | 0.13 | [0.00- 0.81] |  | 5 | 0,26 |  | 0 | 0,00 |  |
| Sarcomatoid carcinoma of lung | **R** | 15 | 0.38 | [0.04- 1.28] |  | 10 | 0,53 |  | 5 | 0,24 |  |
| **EPITHELIAL TUMOURS OF THYMUS** |  |  |  |  |  |  |  |  |  |  |  |
| Malignant thymoma | **R** | 24 | 0.61 | [0.13- 1.65] |  | 14 | 0,74 |  | 10 | 0,48 |  |
| Squamous cell carcinoma of thymus | **R** | 1 | 0.03 | [0.03- 0.58] |  | 1 | 0,05 |  | 0 | 0,00 |  |
| Adenocarcinoma with variants of thymus | **R** | 1 | 0.03 | [0.03- 0.58] |  | 0 | 0,00 |  | 1 | 0,05 |  |
| **MALIGNANT MESOTHELIOMA** |  |  |  |  |  |  |  |  |  |  |  |
| Mesothelioma of pleura and pericardium | **R** | 32 | 0.81 | [0.22- 1.96] |  | 23 | 1,21 |  | 9 | 0,44 |  |
| Mesothelioma of peritoneum and tunica vaginalis | **R** | 5 | 0.13 | [0.00- 0.81] |  | 1 | 0,05 |  | 4 | 0,19 |  |
| **Rare breast** |  | **227** |  |  |  | **0** |  |  | **227** |  |  |
| **EPITHELIAL TUMOURS OF BREAST** |  |  |  |  |  |  |  |  |  |  |  |
| Invasive carcinoma of no special type-NST (obs Invasive ductal carcinoma of breast) |  | 3306 | 83.49 | [75.23- 92.31] |  | 29 | 1,53 |  | 3277 | 158,57 |  |
| Invasive lobular carcinoma of breast |  | 572 | 14.44 | [11.17- 18.29] |  | 0 | 0,00 |  | 572 | 27,68 |  |
| Mammary Paget's disease of breast | **R** | 24 | 0.61 | [0.13- 1.65] |  | 0 | 0,00 |  | 24 | 1,16 |  |
| Special types of adenocarcinoma of breast | **R** | 185 | 4.67 | [2.92- 6.99] |  | 0 | 0,00 |  | 185 | 8,95 |  |
| Metaplastic carcinoma of breast | **R** | 13 | 0.33 | [0.03- 1.19] |  | 0 | 0,00 |  | 13 | 0,63 |  |
| Salivary gland type tumour of breast | **R** | 5 | 0.13 | [0.00- 0.81] |  | 0 | 0,00 |  | 5 | 0,24 |  |
| **Rare female genital** |  | **346** |  |  |  |  |  |  | **346** |  |  |
| Supplementary Table 1. Cont. Incidence rates for rare cancers in all adults, and by sex, from 2011-2020 in Geneva, Switzerland | | | | | | | | | | |  |
| **Cancer sites** | **Total adult population** | | | |  | **Men** | |  | **Women** | |  |
|  | **Rare** | **N** | **Crude rate** | **95% CI** |  | **N** | **Crude rate** |  | **N** | **Crude rate** |  |
| **EPITHELIAL TUMOURS OF CORPUS UTERI** |  |  |  |  |  |  |  |  |  |  |  |
| Adenocarcinoma with variants of corpus uteri |  | 416 | 10.51 | [7.75- 13.82] |  |  |  |  | 416 | 20,13 |  |
| Squamous cell carcinoma with variants of corpus uteri | **R** | 1 | 0.03 | [0.00- 0.58] |  |  |  |  | 1 | 0,05 |  |
| Clear cell adenocarcinoma, NOS | **R** | 11 | 0.28 | [0.02- 1.10] |  |  |  |  | 11 | 0,53 |  |
| Serous (papillary) carcinoma | **R** | 43 | 1.09 | [0.37- 2.37] |  |  |  |  | 43 | 2,08 |  |
| Mullerian mixed tumour | **R** | 25 | 0.63 | [0.14- 1.69] |  |  |  |  | 25 | 1,21 |  |
| **EPITHELIAL TUMOURS OF CERVIX UTERI** |  |  |  |  |  |  |  |  |  |  |  |
| Squamous cell carcinoma with variants of cervix uteri | **R** | 80 | 2.02 | [0.96- 3.65] |  |  |  |  | 80 | 3,87 |  |
| Adenocarcinoma with variants of cervix uteri | **R** | 32 | 0.81 | [0.22- 1.96] |  |  |  |  | 32 | 1,55 |  |
| **EPITHELIAL TUMOURS OF OVARY AND FALLOPPIAN TUBE** |  |  |  |  |  |  |  |  |  |  |  |
| Adenocarcinoma with variants of ovary |  | 266 | 6.72 | [4,57- 9.43] |  |  |  |  | 266 | 12,87 |  |
| Mucinous adenocarcinoma of ovary | **R** | 20 | 0.51 | [0.09- 1.49] |  |  |  |  | 20 | 0,97 |  |
| Clear cell adenocarcinoma of ovary | **R** | 13 | 0.33 | [0.03- 1.19] |  |  |  |  | 13 | 0,63 |  |
| Primary peritoneal serous/papillary carcinoma | **R** | 3 | 0.08 | [0.00- 0.70] |  |  |  |  | 3 | 0,15 |  |
| Mullerian mixed tumour of ovaryand falloppian tube | **R** | 5 | 0.13 | [0.00- 0.81] |  |  |  |  | 5 | 0,24 |  |
| Adenocarcinoma with variants of fallopian tube | **R** | 31 | 0.78 | [0.21- 1.92] |  |  |  |  | 31 | 1,50 |  |
| **NON EPITHELIAL TUMOURS OF OVARY** |  |  |  |  |  |  |  |  |  |  |  |
| Sex cord tumour of ovary | **R** | 3 | 0.08 | [0.00- 0.70] |  |  |  |  | 3 | 0,15 |  |
| Malignant/Immature teratoma of ovary | **R** | 2 | 0.05 | [0.00- 0.64] |  |  |  |  | 2 | 0,10 |  |
| Germ cell tumour of ovary | **R** | 4 | 0.10 | [0.00- 0.75] |  |  |  |  | 4 | 0,19 |  |
| **EPITHELIAL TUMOURS OF VULVA AND VAGINA** |  |  |  |  |  |  |  |  |  |  |  |
| Squamous cell carcinoma with variants of vulva and vagina | **R** | 65 | 1.64 | [0.71- 3.14] |  |  |  |  | 65 | 3,15 |  |
| Adenocarcinoma with variants of vulva and vagina | **R** | 2 | 0.05 | [0.00- 0.64] |  |  |  |  | 2 | 0,10 |  |
| Paget's disease of vulva and vagina | **R** | 5 | 0.13 | [0.00- 0.81] |  |  |  |  | 5 | 0,24 |  |
| **TROPHOBLASTIC TUMOURS OF PLACENTA** |  |  |  |  |  |  |  |  |  |  |  |
| Choriocarcinoma of placenta | **R** | 1 | 0.03 | [0.00- 0.58] |  |  |  |  | 1 | 0,05 |  |
| **Rare male genital and urogenital** |  | **222** |  |  |  | **222** |  |  |  |  |  |
| **EPITHELIAL TUMOURS OF PROSTATE** |  |  |  |  |  |  |  |  |  |  |  |
| Supplementary Table 1. Cont. Incidence rates for rare cancers in all adults, and by sex, from 2011-2020 in Geneva, Switzerland | | | | | | | | | | |  |
| **Cancer sites** | **Total adult population** | | | |  | **Men** | |  | **Women** | |  |
|  | **Rare** | **N** | **Crude rate** | **95% CI** |  | **N** | **Crude rate** |  | **N** | **Crude rate** |  |
| Adenocarcinoma with variants of prostate |  | 3267 | 82.50 | [74.30- 91.28] |  | 3267 | 172,56 |  |  |  |  |
| Infiltrating duct carcinoma of prostate | **R** | 14 | 0.35 | [0.104 1.23] |  | 14 | 0,74 |  |  |  |  |
| **TESTICULAR AND PARATESTICULAR CANCERS** |  |  |  |  |  |  |  |  |  |  |  |
| Non seminomatous testicular cancer | **R** | 73 | 1.84 | [0.84- 3.41] |  | 73 | 3,86 |  |  |  |  |
| Seminomatous testicular cancer | **R** | 108 | 2.73 | [1.45- 4.57] |  | 108 | 5,70 |  |  |  |  |
| **EPITHELIAL TUMOURS OF PENIS** |  |  |  |  |  |  |  |  |  |  |  |
| Squamous cell carcinoma with variants of penis | **R** | 27 | 0.68 | [0.16- 1.77] |  | 27 | 1,43 |  |  |  |  |
| **Rare urological** |  | **145** |  |  |  | **109** |  |  | **36** |  |  |
| **EPITELIAL TUMOURS OF KIDNEY** |  |  |  |  |  |  |  |  |  |  |  |
| Renal cell carcinoma with variants |  | 486 | 12.27 | [9.28- 15.84] |  | 339 | 17,91 |  | 147 | 7,11 |  |
| **EPITHELIAL TUMOURS OF PELVIS AND URETER** |  |  |  |  |  |  |  |  |  |  |  |
| Transitional cell carcinoma of pelvis and ureter | **R** | 96 | 2.42 | [1.24- 4.18] |  | 68 | 3,59 |  | 28 | 1,35 |  |
| Squamous cell carcinoma with variants of pelvis and ureter | **R** | 2 | 0.05 | [0.00- 0.64] |  | 2 | 0,11 |  | 0 | 0,00 |  |
| Adenocarcinoma with variants of pelvis and ureter | **R** | 1 | 0.03 | [0.00- 0.58] |  | 1 | 0,05 |  | 0 | 0,00 |  |
| **EPITHELIAL TUMOURS OF URETHRA** |  |  |  |  |  |  |  |  |  |  |  |
| Transitional cell carcinoma of urethra | **R** | 9 | 0.23 | [0.01- 1.00] |  | 9 | 0,48 |  | 0 | 0,00 |  |
| Squamous cell carcinoma with variants of urethra | **R** | 1 | 0.03 | [0.00- 0.58] |  | 0 | 0,00 |  | 1 | 0,05 |  |
| Adenocarcinoma with variants of urethra | **R** | 1 | 0.03 | [0.00- 0.58] |  | 1 | 0,05 |  | 0 | 0,00 |  |
| **EPITHELIAL TUMOURS OF BLADDER** |  |  |  |  |  |  |  |  |  |  |  |
| Transitional cell carcinoma of bladder |  | 728 | 18.38 | [14.65- 22.68] |  | 586 | 30,95 |  | 142 | 6,87 |  |
| Squamous cell carcinoma with variants of bladder | **R** | 20 | 0.51 | [0.09- 1.49] |  | 16 | 0,85 |  | 4 | 0,19 |  |
| Adenocarcinoma with variants of bladder | **R** | 15 | 0.38 | [0.04- 1.28] |  | 12 | 0,63 |  | 3 | 0,15 |  |
| **Rare melanoma** |  | **33** |  |  |  | **15** |  |  | **18** |  |  |
| **MALIGNANT SKIN MELANOMA** |  |  |  |  |  |  |  |  |  |  |  |
| Malignant skin melanoma |  | 1640 | 41.42 | [35.68- 47.72] |  | 884 | 46,69 |  | 756 | 36,58 |  |
| **MALIGNANT MELANOMA OF MUCOSA AND EXTRACUTANEOUS** |  |  |  |  |  |  |  |  |  |  |  |
| Malignant melanoma of mucosa and extracutaneous | **R** | 10 | 0.25 | [0.01- 1.05] |  | 3 | 0,16 |  | 7 | 0,34 |  |
| **MALIGNANT MELANOMA OF EYE** |  |  |  |  |  |  |  |  |  |  |  |
| Supplementary Table 1. Cont. Incidence rates for rare cancers in all adults, and by sex, from 2011-2020 in Geneva, Switzerland | | | | | | | | | | |  |
| **Cancer sites** | **Total adult population** | | | |  | **Men** | |  | **Women** | |  |
|  | **Rare** | **N** | **Crude rate** | **95% CI** |  | **N** | **Crude rate** |  | **N** | **Crude rate** |  |
| Malignant melanoma of conjunctiva | **R** | 2 | 0.05 | [0.00- 0.64] |  | 1 | 0,05 |  | 1 | 0,05 |  |
| Malignant melanoma of uvea | **R** | 21 | 0.53 | [0.10- 1.53] |  | 11 | 0,58 |  | 10 | 0,48 |  |
| **Rare skin** |  | **92** |  |  |  | **48** |  |  | **44** |  |  |
| **EPITHELIAL TUMOURS OF SKIN** |  |  |  |  |  |  |  |  |  |  |  |
| Basal cell carcinoma of skin | **R** | 13 | 0.33 | [0.03- 1.19] |  | 9 | 0,48 |  | 4 | 0,19 |  |
| Squamous cell carcinoma with variants of skin |  | 5147 | 129.98 | [119.61- 140.97] |  | 2712 | 143,25 |  | 2435 | 117,83 |  |
| **ADNEXAL CARCINOMAS OF SKIN** |  |  |  |  |  |  |  |  |  |  |  |
| Adnexal carcinoma of skin | **R** | 79 | 2.00 | [0.94- 3.61] |  | 39 | 2,06 |  | 40 | 1,94 |  |
| **Rare sarcomas** |  | **450** |  |  |  | **236** |  |  | **214** |  |  |
| **SOFT TISSUE SARCOMA** |  |  |  |  |  |  |  |  |  |  |  |
| Soft tissue sarcoma of head and neck | **R** | 17 | 0.43 | [0.06- 1.36] |  | 14 | 0,74 |  | 3 | 0,15 |  |
| Soft tissue sarcoma of limbs | **R** | 91 | 2.30 | [1.15- 4.01] |  | 53 | 2,80 |  | 38 | 1,84 |  |
| Soft tissue sarcoma of superficial trunk | **R** | 49 | 1.24 | [0.46- 2.58] |  | 19 | 1,00 |  | 30 | 1,45 |  |
| Soft tissue sarcoma of mediastinum | **R** | 1 | 0.03 | [0.00- 0.58] |  | 0 | 0,00 |  | 1 | 0,05 |  |
| Soft tissue sarcoma of heart | **R** | 3 | 0.08 | [0.00- 0.70] |  | 1 | 0,05 |  | 2 | 0,10 |  |
| Soft tissue sarcoma of breast | **R** | 10 | 0.25 | [0.01- 1.05] |  | 0 | 0,00 |  | 10 | 0,48 |  |
| Soft tissue sarcoma of uterus | **R** | 36 | 0.91 | [0.27- 2.11] |  | 0 | 0,00 |  | 36 | 1,74 |  |
| Soft tissue sarcoma of paratestis | **R** | 4 | 0.10 | [0.00- 0.75] |  | 4 | 0,21 |  | 0 | 0,00 |  |
| Soft tissue sarcomas of other genitourinary tract (vulva, vagina, ovary, penis, prostate, testis, kidney, renal pelvis, ureter, bladder, urethra) | **R** | 8 | 0.20 | [0.01- 0.96] |  | 3 | 0,16 |  | 5 | 0,24 |  |
| Soft tissue sarcoma of viscera | **R** | 16 | 0.40 | [0.05- 1.32] |  | 8 | 0,42 |  | 8 | 0,39 |  |
| Soft tissue sarcoma of retroperitoneum and peritoneum | **R** | 17 | 0.43 | [0.06- 1.36] |  | 9 | 0,48 |  | 8 | 0,39 |  |
| Soft tissue sarcoma of pelvis | **R** | 12 | 0.30 | [0.02- 1.14] |  | 9 | 0,48 |  | 3 | 0,15 |  |
| Soft tissue sarcoma of skin | **R** | 24 | 0.61 | [0.13- 1.65] |  | 15 | 0,79 |  | 9 | 0,44 |  |
| Soft tissue sarcoma of brain and other parts of the nervous system | **R** | 4 | 0.10 | [0.00- 0.75] |  | 2 | 0,11 |  | 2 | 0,10 |  |
| Embryonal rhabdomyosarcoma of soft tissue | **R** | 1 | 0.03 | [0.00- 0.58] |  | 0 | 0,00 |  | 1 | 0,05 |  |
| Ewing's sarcoma of soft tissue | **R** | 2 | 0.05 | [0.00- 0.64] |  | 0 | 0,00 |  | 2 | 0,10 |  |
| **BONE SARCOMA** |  |  |  |  |  |  |  |  |  |  |  |
| Supplementary Table 1. Cont. Incidence rates for rare cancers in all adults, and by sex, from 2011-2020 in Geneva, Switzerland | | | | | | | | | | |  |
| **Cancer sites** | **Total adult population** | | | |  | **Men** | |  | **Women** | |  |
|  | **Rare** | **N** | **Crude rate** | **95% CI** |  | **N** | **Crude rate** |  | **N** | **Crude rate** |  |
| Osteogenic sarcoma | **R** | 3 | 0.08 | [0.00- 0.70] |  | 1 | 0,05 |  | 2 | 0,10 |  |
| Chondrogenic sarcoma | **R** | 26 | 0.66 | [0.15- 1.73] |  | 15 | 0,79 |  | 11 | 0,53 |  |
| Notochordal sarcoma, chordoma | **R** | 7 | 0.18 | [0.00- 0.91] |  | 6 | 0,32 |  | 1 | 0,05 |  |
| Ewing's sarcoma | **R** | 5 | 0.13 | [0.00- 0.81] |  | 1 | 0,05 |  | 4 | 0,19 |  |
| **GASTROINTESTINAL STROMAL SARCOMA** |  |  |  |  |  |  |  |  |  |  |  |
| Gastrointestinal stromal sarcoma | **R** | 69 | 1.74 | [0.77- 3.28] |  | 34 | 1,80 |  | 35 | 1,69 |  |
| **KAPOSI'S SARCOMA** |  |  |  |  |  |  |  |  |  |  |  |
| Kaposi's sarcoma | **R** | 45 | 1.14 | [0.40- 2.44] |  | 42 | 2,22 |  | 3 | 0,15 |  |
| **Rare endocrine and neuroendocrine** |  | **465** |  |  |  | **219** |  |  | **246** |  |  |
| **NET LUNG** |  |  |  |  |  |  |  |  |  |  |  |
| Typical and atypical carcinoid of the lung | **R** | 69 | 1.74 | [0.77- 3.28] |  | 27 | 1,43 |  | 42 | 2,03 |  |
| **NET GEP** |  |  |  |  |  |  |  |  |  |  |  |
| Well differentiated not functioning endocrine carcinoma of pancreas and digestive tract | **R** | 190 | 4.80 | [3.02- 7.14] |  | 85 | 4,49 |  | 105 | 5,08 |  |
| Poorly differentiated endocrine carcinoma of pancreas and digestive tract | **R** | 87 | 2.20 | [1.08- 3.88] |  | 38 | 2,01 |  | 49 | 2,37 |  |
| Malignant mixed pancreatic endocrine and exocrine tumour | **R** | 2 | 0.05 | [0.00- 0.64] |  | 2 | 0,11 |  | 0 | 0,00 |  |
| **NET OTHER SITES** |  |  |  |  |  |  |  |  |  |  |  |
| Pheochromocytoma, malignant | **R** | 3 | 0.08 | [0.00- 0.70] |  | 2 | 0,11 |  | 1 | 0,05 |  |
| Paraganglioma | **R** | 2 | 0.05 | [0.00- 0.64] |  | 2 | 0,11 |  | 0 | 0,00 |  |
| Endocrine carcinoma of thyroid gland | **R** | 12 | 0.30 | [0.02- 1.14] |  | 7 | 0,37 |  | 5 | 0,24 |  |
| Neuroendocrine carcinoma of skin | **R** | 25 | 0.63 | [0.14- 1.69] |  | 15 | 0,79 |  | 10 | 0,48 |  |
| Neuroendocrine carcinoma of other sites | **R** | 62 | 1.57 | [0.66- 3.04] |  | 36 | 1,90 |  | 26 | 1,26 |  |
| **CARCINOMAS OF THYROID GLAND** |  |  |  |  |  |  |  |  |  |  |  |
| Carcinoma of thyroid gland |  | 701 | 17.70 | [14.05- 21.92] |  | 161 | 8,50 |  | 540 | 26,13 |  |
| **CARCINOMAS OF PARATHYROID GLAND** |  |  |  |  |  |  |  |  |  |  |  |
| Carcinoma of parathyroid gland | **R** | 4 | 0.10 | [0.00- 0.75] |  | 0 | 0,00 |  | 4 | 0,19 |  |
| **CARCINOMAS OF ADRENAL CORTEX** |  |  |  |  |  |  |  |  |  |  |  |
| Carcinoma of adrenal cortex | **R** | 9 | 0.23 | [0.01- 1.00] |  | 5 | 0,26 |  | 4 | 0,19 |  |
| **CNS** |  | **46** |  |  |  | **24** |  |  | **22** |  |  |
| Supplementary Table 1. Cont. Incidence rates for rare cancers in all adults, and by sex, from 2011-2020 in Geneva, Switzerland | | | | | | | | | | |  |
| **Cancer sites** | **Total adult population** | | | |  | **Men** | |  | **Women** | |  |
|  | **Rare** | **N** | **Crude rate** | **95% CI** |  | **N** | **Crude rate** |  | **N** | **Crude rate** |  |
| **TUMOURS OF CENTRAL NERVOUS SYSTEM (CNS)** |  |  |  |  |  |  |  |  |  |  |  |
| Astrocytic tumours of CNS |  | 240 | 6.06 | [4.03- 8.65] |  | 152 | 8,03 |  | 88 | 4,26 |  |
| Oligodendroglial tumours of CNS | **R** | 26 | 0.66 | [0.15- 1.73] |  | 16 | 0,85 |  | 10 | 0,48 |  |
| Ependymal tumours of CNS SANS /1 | **R** | 12 | 0.30 | [0.02- 1.14] |  | 5 | 0,26 |  | 7 | 0,34 |  |
| Malignant meningiomas | **R** | 4 | 0.10 | [0.00- 0.75] |  | 2 | 0,11 |  | 2 | 0,10 |  |
| Tumours of the pineal gland | **R** | 1 | 0.03 | [0.00- 0.58] |  | 0 | 0,00 |  | 1 | 0,05 |  |
| **EMBRYONAL TUMOURS OF CNS** |  |  |  |  |  |  |  |  |  |  |  |
| Embryonal tumour of CNS | **R** | 3 | 0.08 | [0.00- 0.70] |  | 1 | 0,05 |  | 2 | 0,10 |  |
| **Rare hematologic** |  | **1565** |  |  |  | **833** |  |  | **732** |  |  |
| **LYMPHOID DISEASES** |  |  |  |  |  |  |  |  |  |  |  |
| Hodgkin lymphoma, classical | **R** | 142 | 3.59 | [2.09- 5.65] |  | 81 | 4,28 |  | 61 | 2,95 |  |
| Hodgkin lymphoma nodular lymphocyte predominance | **R** | 9 | 0.23 | [0.01- 1.00] |  | 6 | 0,32 |  | 3 | 0,15 |  |
| Precursor B/T lymphoblastic leukaemia/lymphoblastic lymphoma (and Burkitt leukemia/lymphoma) | **R** | 35 | 0.88 | [0.26- 2.07] |  | 18 | 0,95 |  | 17 | 0,82 |  |
| T cutaneous lymphoma (Sezary syn, Mycosis fung) | **R** | 64 | 1.62 | [0.69- 3.11] |  | 36 | 1,90 |  | 28 | 1,35 |  |
| Other T cell lymphomas and NK cell neoplasms | **R** | 61 | 1.54 | [0.64- 3.00] |  | 30 | 1,58 |  | 31 | 1,50 |  |
| Diffuse B lymphoma |  | 395 | 9.98 | [7.30- 13.22] |  | 211 | 11,14 |  | 184 | 8,90 |  |
| Follicular B lymphoma | **R** | 170 | 4.29 | [2.63- 6.52] |  | 84 | 4,44 |  | 86 | 4,16 |  |
| Hairy cell leukaemia | **R** | 23 | 0.58 | [0.12- 1.61] |  | 21 | 1,11 |  | 2 | 0,10 |  |
| Plasmacytoma/Multiple Myeloma (and Heavy chain diseases) |  | 318 | 8.03 | [5.66- 10.97] |  | 163 | 8,61 |  | 155 | 7,50 |  |
| Other non Hodgkin, Mature B cell lymphoma |  | 615 | 15.53 | [12.12- 19.50] |  | 345 | 18,22 |  | 270 | 13,06 |  |
| Mantle cell lymphoma | **R** | 66 | 1.67 | [0.72- 3.17] |  | 47 | 2,48 |  | 19 | 0,92 |  |
| Prolymphocytic leukaemia, B cell | **R** | 5 | 0.13 | [0.00- 0.81] |  | 2 | 0,11 |  | 3 | 0,15 |  |
| **ACUTE MYELOID LEUKEMIA AND RELATED PRECURSOR NEOPLASMS** |  |  |  |  |  |  |  |  |  |  |  |
| Acute promyelocytic leukemia (AML with t(15;17) with variants | **R** | 15 | 0.38 | [0.04- 1.28] |  | 6 | 0,32 |  | 9 | 0,44 |  |
| AML | **R** | 201 | 5.08 | [3.24- 7.47] |  | 102 | 5,39 |  | 99 | 4,79 |  |
| **MYELOID AND LYMPHOID NEOPLASMS** |  |  |  |  |  |  |  |  |  |  |  |
| Myeloid and lymphoid neoplasms | **R** | 5 | 0.13 | [0.00- 0.81] |  | 2 | 0,11 |  | 3 | 0,15 |  |
| **MYELOPROLIFERATIVE NEOPLASMS** |  |  |  |  |  |  |  |  |  |  |  |
| Supplementary Table 1. Cont. Incidence rates for rare cancers in all adults, and by sex, from 2011-2020 in Geneva, Switzerland | | | | | | | | | | |  |
| **Cancer sites** | **Total adult population** | | | |  | **Men** | |  | **Women** | |  |
|  | **Rare** | **N** | **Crude rate** | **95% CI** |  | **N** | **Crude rate** |  | **N** | **Crude rate** |  |
| Chronic myeloid leukemia | **R** | 60 | 1.52 | [0.63- 2.97] |  | 35 | 1,85 |  | 25 | 1,21 |  |
| Other myeloproliferative neoplasms |  | 244 | 6.16 | [4.11- 8.77] |  | 116 | 6,13 |  | 128 | 6,19 |  |
| Mast cell tumour | **R** | 3 | 0.08 | [0.00- 0.70] |  | 1 | 0,05 |  | 2 | 0,10 |  |
| **MYELODYSPLASTIC SYNDROME AND MYELODYSPLASTIC/MYELOPROLIFERATIVE DISEASES** | | |  |  |  |  |  |  |  |  |  |
| Myelodysplastic syndrome with 5q syndrome | **R** | 5 | 0.13 | [0.00- 0.81] |  | 1 | 0,05 |  | 4 | 0,19 |  |
| Other myelodysplastic syndrome |  | 294 | 7.42 | [5.15- 10.26] |  | 176 | 9,30 |  | 118 | 5,71 |  |
| Chronic Myelomonocytic leukemia | **R** | 56 | 1.41 | [0.56- 2.83] |  | 32 | 1,69 |  | 24 | 1,16 |  |
| **HISTIOCYTIC AND DENDRITIC CELL NEOPLASMS** |  |  |  |  |  |  |  |  |  |  |  |
| Histiocytic malignancies | **R** | 6 | 0.15 | [0.00- 0.86] |  | 2 | 0,11 |  | 4 | 0,19 |  |
| **Other** |  | **15** |  |  |  | **9** |  |  | **6** |  |  |
| **OLFACTORY NEUROBLASTOMA** |  |  |  |  |  |  |  |  |  |  |  |
| Olfactory neuroblastoma | **R** | 1 | 0.03 | [0.00- 0.58] |  | 0 | 0,00 |  | 1 | 0,05 |  |
| **EXTRAGONADAL GERM CELL TUMOURS** |  |  |  |  |  |  |  |  |  |  |  |
| Non seminomatous germ cell tumour | **R** | 8 | 0.20 | [0.01- 0.96] |  | 7 | 0,37 |  | 1 | 0,05 |  |
| Seminomatous germ cell tumor | **R** | 1 | 0.03 | [0.00- 0.58] |  | 1 | 0,05 |  | 0 | 0,00 |  |
| **EPITHELIAL TUMOURS OF EYE AND ADNEXA** |  |  |  |  |  |  |  |  |  |  |  |
| Squamous cell carcinoma with variants of eye and adnexa | **R** | 5 | 0.13 | [0.00- 0.81] |  | 1 | 0,05 |  | 4 | 0,19 |  |

**Abbrevations**: **R**, rare as per <6/100’000 **N**, Number of cases; **Crude rate,** incidence rate per 100'000; **95% CI**, 95% Confidence Interval. Incidence rates were standardized to the 1976 European reference population

**Classification**: ***** corresponds to the major families of rare cancers; ****** corresponds to the major clinically relevant entities, Tier 1 entities; ******* Tier 1 entities are broken down into Tier 2 entities which are distinguished by their morphologies and topographies. The asterisks have been added for only one example to designate a family of rare cancers such as “head and neck”; an entity corresponding to a Tier 1 category such as “epithelial tumors of the nasal cavities and sinuses”; and a Tier 2 entity such as “squamous cell carcinoma with variants of the nasal cavities and sinuses”.
